# Changes in the SCM response ratio (RRscm) after surgical removal of malignant tissue.

**DOI:** 10.1038/bjc.1975.32

**Published:** 1975-02

**Authors:** L. Cercek, B. Cercek


Br. J. Cancer (1975) 31, 250

### Short Communication

### CHANGES IN THE SCM RESPONSE RATIO ($RR_{SCM}$) AFTER SURGICAL REMOVAL OF MALIGNANT TISSUE

L. CERCEK AND B. CERCEK

*From the Paterson Laboratories, Christie Hospital and Holt Radium Institute, Manchester, M20 9BX*

Received 28 October 1974. Accepted 18 November 1974

IN OUR recent report, we have shown that lymphocytes from patients with malignant disorders can be differentiated from those of healthy donors or donors with non-malignant disorders on the basis of changes in the structuredness of cytoplasmic matrix (SCM) induced by cancer basic protein (CaBP) and phytohaemagglutinin (PHA) (Cercek, Cercek and Franklin, 1974). Changes in the SCM are measured by means of the technique of fluorescence polarization (Cercek, Cercek and Ockey, 1973). To increase the resolution of the SCM test and to assess the general immunocompetence of the donor's lymphocytes, we have expressed the SCM response of lymphocytes to CaBP and to PHA as a single parameter, *i.e.* SCM Response Ratio ($RR_{SCM}$):

$$RR_{SCM} = P_{CaBP}/P_{PHA}$$

where $P_{CaBP}$ means the degree of fluorescence polarization obtained after CaBP stimulation and $P_{PHA}$ that after PHA stimulation, both measured at comparable times after stimulation. Details of the technique have already been described (Cercek *et al.*, 1974).

The values of $RR_{SCM}$ of lymphocytes from patients with malignant disorders range from 0·6 to 1·0 whereas $RR_{SCM}$ values of lymphocytes from healthy donors or donors with non-malignant disorders range from 1·2 to 1·7 (Cercek *et al.*, 1974). We have now studied changes in $RR_{SCM}$ values after surgical removal of malignant tissues. The $RR_{SCM}$ values were assessed before operation, 24 h after operation and 2 weeks later. Results in Table I show that there was a progressive increase in the values of $RR_{SCM}$ after surgery and in 5/6 of these cases reached values typical for healthy donors 2 weeks after operation.

After removal of the malignant tissue, lymphocytes first lost the ability to respond to CaBP and 2 weeks later they regained the ability to respond to PHA. The absence of the response to CaBP and PHA 24 h after surgery is not due to an effect of anaesthetics as we have found that after surgery in non-malignant disorders there was no change in the values of $RR_{SCM}$ before and after operation. The loss of the response to CaBP in 24 h after removal of malignant tissues seems to suggest that the half-life of receptors for CaBP on lymphocytes from patients with cancer is not longer than the order of hours.

We have also studied changes in $RR_{SCM}$ values before and after surgical removal or biopsies of histologically declared benign growths in the breast. The results in Table II show that 4/12 cases did not respond to CaBP and PHA and could on this basis be benign or pre-malignant (Cercek *et al.*, 1974). However, in 8 cases lymphocytes responded to CaBP and gave no response to PHA. The $RR_{SCM}$ values were typical for malignant conditions. Also, their recovery pattern after operation was the same as in

TABLE I.—*Changes in $RR_{SCM}$ after Surgical Removal of Malignant Tissues*

| Site of malignancy | Donor's Age | Donor's Sex | 24 h pre-operation | 24 h post-operation | 2 weeks post-operation |
|---|---|---|---|---|---|
| Colon | 72 | M | 0·83 | 1·02 | 1·20 |
| Colon | 77 | F | 0·85 | 1·05 | 1·29 |
| Breast | 73 | F | 0·84 | 0·97 | 1·03 |
| Breast | 37 | F | 0·76 | 1·01 | 1·20 |
| Breast | 54 | F | 0·83 | 1·02 | 1·23 |
| Breast | 51 | F | 0·89 | 0·99 | 1·20 |

TABLE II.—*Changes in $RR_{SCM}$ after Surgical Removal of Histologically Declared Benign Growth in the Breast*

| Donor's Age | Donor's Sex | 24 h pre-operation | 24 h post-operation | 2 weeks post-operation |
|---|---|---|---|---|
| 38 | F | 1·01* | 0·99 | 1·01 |
| 45 | F | 0·92* | 0·91 | 1·11 |
| 31 | F | 0·99* | 0·86 | 1·05 |
| 64 | M | 0·96* | 0·84 | 1·22 |
| 40 | F | 0·78 | 1·02 | 1·19 |
| 25 | F | 0·84 | 0·97 | 1·19 |
| 52 | F | 0·81 | 1·01 | 1·15 |
| 32 | F | 0·78 | 1·10 | — |
| 45 | F | 0·79 | 0·98 | 1·27 |
| 43 | F | 0·85 | 0·98 | 1·15 |
| 45 | F | 0·80 | 1·02 | 1·00 |
| 54 | F | 0·87 | 0·87 | 0·95 |

* No response to CaBP.

histologically declared malignant growths. The results raise the possibility that the sensitization to CaBP indicates cases which may be committed to become histologically recognizable malignancies if the growths were left *in situ*, and only cases whose lymphocytes did not recognize CaBP may represent genuine benign growths. The fact that they also did not respond to PHA places them into a similar category of the previously reported 3 " pre-malignant " cases (Cercek and Cercek, 1974). They are in contrast to cases of benign pituitary tumours, lipomata and benign growth of prostate which did not respond to CaBP and gave a normal response to PHA, *i.e.* $RR_{SCM}$ values greater than 1·2 (Cercek and Cercek, 1974). More data on benign cases are being collected.

In summary, this study indicates that changes in $RR_{SCM}$ values after surgery may become a useful aid to check if the malignant growth has been removed successfully and to monitor possible recurrence of the disease.

We thank the surgeons of the Teaching Unit 2 of the Withington Hospital and the surgeons of the Professional Surgical Unit of the University Hospital of South Manchester for their collaboration. This work was supported by grants from the Cancer Research Campaign and the Medical Research Council.

### REFERENCES

CERCEK, L., CERCEK, B. & OCKEY, C. H. (1973) Structuredness of the Cytoplasmic Matrix and Michaelis-Menten Constants for the Hydrolysis of FDA during the Cell Cycle in Chinese Hamster Ovary Cells. *Biophysik*, **10**, 187.

CERCEK, L., CERCEK, B. & FRANKLIN, C. I. V. (1974) Biophysical Differentiation Between Lymphocytes from Healthy Donors, Patients with Malignant Diseases and Other Disorders. *Br. J. Cancer*, **29**, 345.

CERCEK, L. & CERCEK, B. (1974) Changes in the Structuredness of Cytoplasmic Matrix of Lymphocytes as a Diagnostic and Prognostic Test for Cancer. In *Proc. XIth Internat. Cancer Cong.*, Florence. 20–26 October 1974, Italy. In the press.


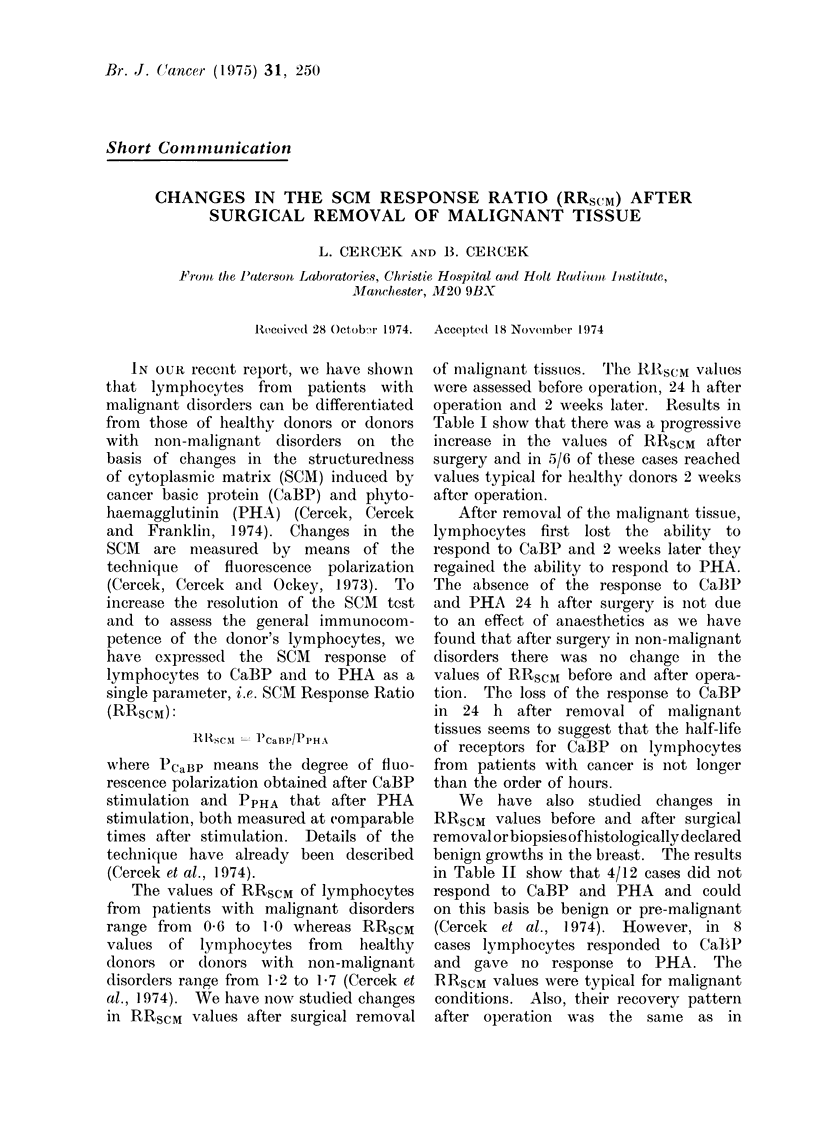

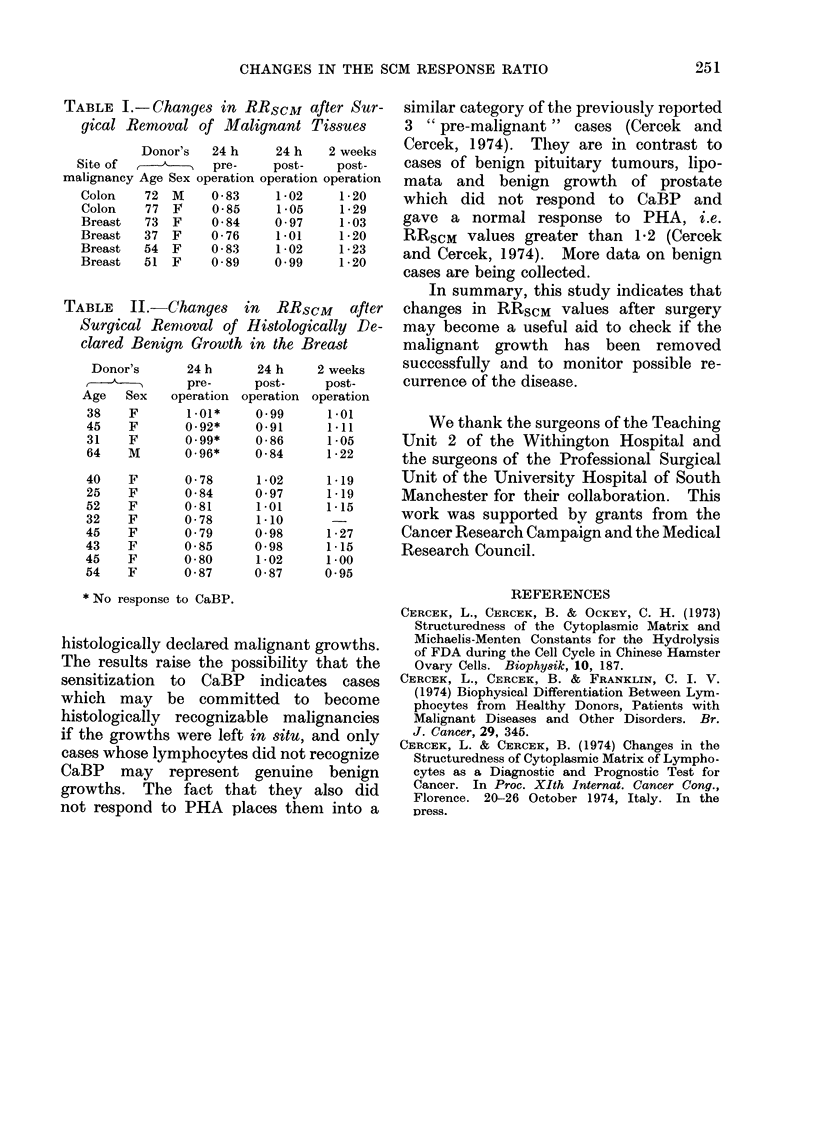


## References

[OCR_00189] Cercek L., Cercek B., Franklin C. I. (1974). Biophysical differentiation between lymphocytes from healthy donors, patients with malignant diseases and other disorders.. Br J Cancer.

[OCR_00182] Cercek L., Cercek B., Ockey C. H. (1973). Structuredness of the cytoplasmic matrix and Michaelis-Menten constants for the hydrolysis of FDA during the cell cycle in Chinese hamster ovary cells.. Biophysik.

[OCR_00002] Kirklin B. R. (1929). THE ROENTGEN RAY IN THE DIAGNOSIS OF PRIMARY CARCINOMA OF THE LUNG.. Cal West Med.

